# Notes from Australia

**Published:** 1898-06-25

**Authors:** 


### NOTES FROM AUSTRALIA.

### THE CHILDREN'S HOSPITAL, MELBOURNE.

The committee of the Hospital for Sick Children are about to embark on very extensive building operations. The additions are to include a new out-patients' department, kitchen block, steam laundry and disinfector, and mortuary, with sewerage and sanitary plumbing, new roads and fencing. The whole is calculated to cost at least £16,000. The foundation-stone of the new buildings was laid on April 20th with very pretty ceremony by three little girls, chosen as representative of the children of Victoria; the three were—Miss Beatrice Marie St. John Madden, youngest child of Sir John Madden, Chief Justice, and at present Acting Governor of Victoria; Miss Ivy Victoria Clarke, youngest child of the late Sir William Clarke, the first Victorian Baronet; and Miss Ethel Marie Sumner Ryan, only child of Dr. Charles Ryan, who went through the Turkish and Roumanian war, and granddaughter of Mrs. Sumner, the greatest benefactor the Children's Hospital has ever had. The children acquitted themselves well in the presence of a large and distinguished company, that included the Acting-Governor and Lady Madden. The collection on the foundation-stone did not come up to expectations, as it only reached the sum of £180; but Victoria cannot yet be said to have recovered from its financial storm. The committee and the energetic honorary secretary, Mrs. C. H. Nicolson, however, are quite sanguine of being able to make ends meet.

